# Methods and measures in food service food safety research: A review of the published literature

**DOI:** 10.1016/j.heliyon.2024.e25798

**Published:** 2024-02-10

**Authors:** Veronika Bulochova, Ellen W. Evans, Claire Haven-Tang, Elizabeth C. Redmond

**Affiliations:** a ZERO2FIVE Food Industry Centre, Cardiff Metropolitan University, Cardiff, United Kingdom; b Welsh Centre for Tourism Research, Cardiff School of Management, Cardiff Metropolitan University, Cardiff, United Kingdom

**Keywords:** Food service, Food safety, Food handlers, Research methods, Hospitality, Food safety practices, Cognition, Behaviour

## Abstract

**Purpose:**

Food handlers working in food service establishments need to perform appropriate and consistent food safety practices to ensure that the prepared food is safe for consumers to eat, as a failure to do so may lead to foodborne illness. It is important to have an in-depth understanding of food handler food safety behaviour in the food service sector to design targeted educational interventions and approaches to safeguard consumers. The purpose of this review is to provide insight to the existing body of research regarding food handler food safety in the food service sector, and to identify future research needs in terms of methods and measures.

**Methods:**

This study is a review of published food safety research studies (*n* = 118) focusing on food handlers in the food service sector conducted between 2001 and 2021. Data were collated regarding the methods used and the measures assessed in published studies to identify the gaps in current knowledge related to food safety in food service.

**Findings:**

The majority of studies were conducted in the United States (29%), Brazil (17%) and United Kingdom (7%). A large proportion were carried out in restaurants (70%). Less than a third of studies (28%) relied on a mixed method approach for data collection; and only 12% of studies assessed a combination of cognitive and behavioural measures. Observation was used in only 29% of studies. A lack of observational data detailing food safety practices such as handwashing, temperature control, separation and cleaning was determined.

**Significance:**

Such findings indicate a greater need for mixed method approach in future food safety research. It is recommended that the understanding of food handler food safety performance is best obtained through a combined assessment of cognitive and behavioural measures with subsequent triangulation of findings to reduce bias and to obtain reliable results.

## Highlights

• Methods and measures in 118 food service food safety studies were investigated.
• Gaps were identified related to specific food safety findings.
• Limited use of a mixed method and mixed measure approaches in reviewed studies.
• Assessment of cognition and behaviour in combination is needed in future research.
• Triangulation of findings is recommended to obtain in-depth understanding.

## Graphical abstract

**Figure:**
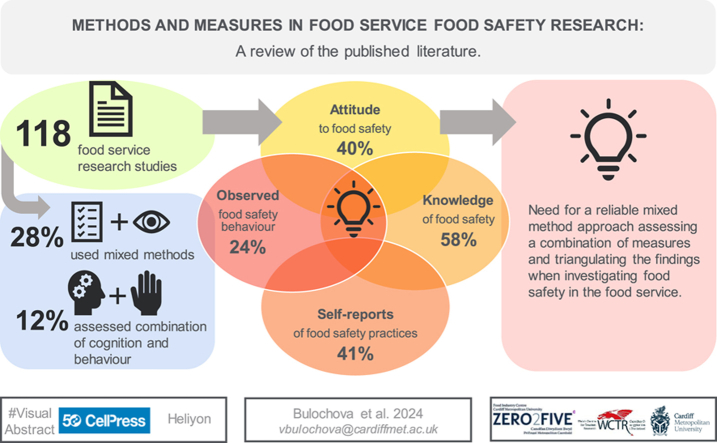

## 1 Introduction

At the final stage of food production and service, food handlers play a vital role in ensuring the safety of food prepared in food service establishments. Worldwide the number of people consuming food prepared out of home is reported to be increasing, driven by urbanization [1]. However, in Europe over a third of foodborne illness outbreaks are reported to be attributed to public eating places such as restaurants, take-aways, street vendors and canteens [2]. Moreover, in US restaurants, a lack of food safety practices during handling and preparation was found to be the most commonly reported contributing factor associated with outbreaks of foodborne illness [3].

### 1.1 Food safety guidance and food safety compliance

To reduce the risk of foodborne illness in the food service sector ‘best practice’ guidelines have been designed by the health authorities and government agencies worldwide, highlighting the importance of appropriate food safety practices: preventing cross-contamination, hand hygiene, cleaning and disinfecting, temperature control when cooking, chilling, hot-holding defrosting and storing food, separation of raw and cooked foods, and using of safe water and raw materials [4,5].

However, food handler food safety compliance is complex and involves multiple factors and, despite the legal requirements and guidance for recommended best practice, foodborne illness cases in food service establishments remain a concern [6,7]. Therefore, gaining understanding of the various behavioural and cognitive influences that may affect food handlers’ food safety compliance and how they interact is central to designing targeted approaches for improvement of food safety in food service establishments.

### 1.2 Food service food handler research

Numerous research studies have been published in the last 20 years with the purpose of ascertaining how food service employees handle food in food service establishments. However, it is important to consider what approaches were used to assess food handler behaviour and cognition and whether the findings reflect the real-life scenario. Moreover, it is important for the data collection instruments to be valid, reliable and applicable, considering the complexity of food service sector.

Few previous reviews explored research methods and measures in the context of food safety [8,9]. Other previous reviews have discussed a range of various topics related to food safety in food service, such as motivational models and behaviour interventions to improve hand hygiene among food service employees [10,11]; food safety behavioural intentions of food handlers [12]; efficacy of food safety training and training interventions [[13], [14], [15]] and the importance of food safety culture [16].

### 1.3 Purpose of this review

In contrast to previous reviews, the aim of this review was to explore the methods and measures in previously published peer-reviewed research (2001–2021) and to provide insight to the current body of knowledge regarding food handler food safety cognition and behaviour, whilst also highlighting areas in which data are lacking. This review aimed to suggest opportunities for future research related specifically to food service food safety behaviour.

## 2 Material and methods

This review was conducted by one researcher following a protocol agreed by the research team. To achieve the aims, elements of a systematic review were used for literature identification [17]. The approach used in this study facilitated a systematic discovery of a wide range of published articles, focused on food safety in the food service sector; whilst also allowing the exploration of various study characteristics to be informed by the content of the collated literature. Ethical Approval for this review was obtained from Cardiff School of Sport and Health Sciences Ethics Committee (Project Reference: PGR-4434).

### 2.1 Population of interest

The review was focused on the population which included food handlers employed in food service establishments (public: restaurants, cafes, fast food, hotels; and institutional: hospital canteens, care home kitchens, school canteens). In this review the term ‘food handlers’ refers to *“people who directly touch open food as part of their work”*. However, it also includes *“anyone who may touch food contact surfaces or other surfaces in rooms where open food is handled”* [18]. Therefore, this term refers not only to chefs, but also to waiters, catering assistants, baristas, food service managers and other food service employees.

### 2.2 Literature search

**Fig. 1:**
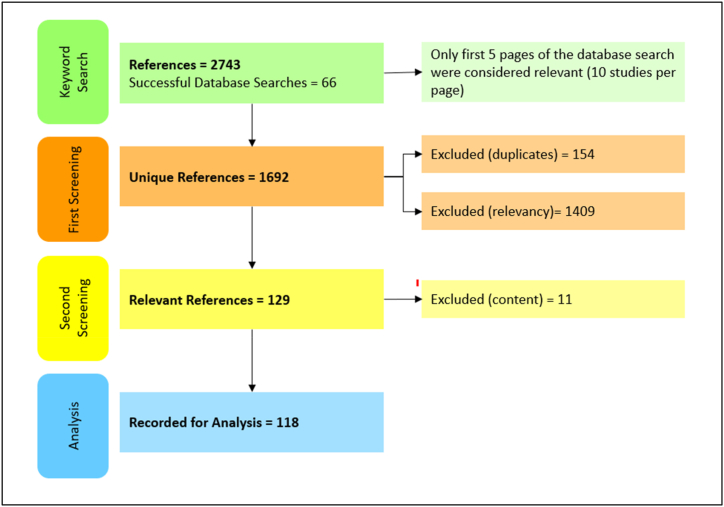
The search flow chart, describing literature search, screening and record.

A systematic search of peer reviewed published articles was conducted in the period of July–September 2021 using the ‘Met Search’ literature search engine by Cardiff Metropolitan University (see Fig. 1). The engine combines the search output from various databases, such as ProQuest Central, PubMed Central, Scopus, Elsevier Science Direct Journals, Wiley Online Library and others [19]. The keywords used in published food safety literature and related to food handler food safety behaviour were utilised for the pilot search and later refined for the purposes of the main search. Final keyword combinations included following categories: general (e.g., food safety, food hygiene), setting (e.g., restaurant, hospitality, food service, catering), measure (e.g., behaviour, compliance, handwashing), person (e.g., food handler, employee, manager, chef). Keywords were used in combinations of two or three, always including the ‘general’ category keyword and resulted in 66 successful search attempts.

The advanced search criteria specified that the articles were peer-reviewed, in English, and published within the last 20 years (from January 2001–October 2021). This time period was chosen to collect data which is relatively recent and could provide an adequate literature sample. The search criteria also specified that the studies are published in English, therefore studies in other languages were not included in this review.

### 2.3 Screening process

The abstracts of studies obtained through the search were initially screened for relevance; subsequently full text PDF files were saved chronologically (Fig. 1). The main inclusion criteria specified that the article must: be focused on the topic of food safety; describe a food service setting (public: restaurants, cafes, fast food, hotels; institutional: hospital canteens, care home kitchens, school and university canteens); involve food handlers or food service managers. Studies describing food service settings not located in a designated building or premises, such as mobile catering, events, or food stalls, were not included in this review. Only original research studies were considered for this review; review studies were excluded. As the purpose of this review was to explore and synthesise all available peer-reviewed findings, it is important to acknowledge that the risk-of-bias assessment and an in-depth critical appraisal of the individual studies included in this review were not completed prior to the data extraction.

### 2.4 Data extraction

A bespoke database was created specifically for the data collation using Qualtrics Software (Qualtrics XM, Provo, UT, USA). The database allowed capture of the key quantitative and qualitative features of collated literature and characteristics relevant to the topic of the study such as: publication details (year, authors, abstracts), study methodology (data collection instruments and methods), behavioural and cognitive measures (knowledge, attitude, self-reports and observational data). This record was performed chronologically. Overall details and data from 118 studies were recorded into the database for further analysis.

### 2.5 Data review and analysis

All captured quantitative data were exported from the Qualtrics database and analysed using Microsoft Excel (Microsoft, Redmond, WA, USA), utilising tools for descriptive statistics. Study measures were compared to enable identification of the gaps in the collated literature. Methods, described in the reviewed studies, were evaluated to determine which approaches were utilised the most and their application, considering the complexity of food service environment.

## 3 Results and discussion

In this review, peer-reviewed research studies (*n* = 118) over the past 20 years detailing food handler food safety were collated and assessed. Main characteristics of the studies, methodology and themes were consolidated to establish current knowledge and to identify gaps regarding food handler food safety in food service sector, as seen in Table 1.

**Table 1:** Characteristics of the reviewed food service food safety studies (*n* = 118).

Characteristic	No	%
**Years of publication**		
2019–2021	40	34
2015–2018	33	28
2011–2014	23	19
2007–2010	17	14
2003–2006	5	4
**Countries of origin**^a^		
United States	34	29
Brazil	20	17
United Kingdom	8	7
Malaysia	6	5
Ethiopia	4	3
**Types of food service settings**^b^		
Restaurant/café/pub	83	70
School/University	37	31
Care service/Hospital	26	22
Hotel/B&B/Guest House	21	18
Takeaway/Sandwich Shop	20	17
Single food service setting	79	67
Multiple food service setting	39	33
**Methodology**		
Single method used for data collection	85	72
Mixed methods used for data collection	33	28
**Themes and measures investigated**^b^		
Knowledge	69	58
Self-reported practices	48	41
Attitudes	47	40
Observed Behaviour	28	24
Food safety culture	20	17
Perceptions of risk/susceptibility	15	13
Microbiological analysis results	12	10
Barriers to compliance	9	8
**Population investigated**		
Food service personnel	116	98
Food service personnel and consumers	2	2

a Additional countries of origin where three studies were sourced were: China; Ghana; Spain; Turkey; United Arab Emirates; where two studies were sourced included: Canada; Ireland; Italy; Jordan; Kuwait; Lebanon; New Zealand; Nigeria; South Africa; Taiwan; where one study was sourced included: Austria; Bangladesh; Egypt; India; Korea, South; Oman; Portugal; Qatar; Saudi Arabia; Thailand; Vietnam; Zimbabwe.

b Multiple selections were possible, so percentage will not add up to 100%.

### 3.1 Years of publication and the origins of food safety studies

Although the literature search was performed online for the time period between the years 2001–2021, the earliest study obtained for this review was undertaken in 2003 and the most recent in 2021 (Table 1). A third (34%) of all studies identified were conducted between 2019 and 2021, indicating increased attention to the topic of food safety in food service establishments in recent years. Cultural changes, busy consumer lifestyle and the availability of cheaper food options has facilitated a change in consumer preference towards eating out of home; this in turn has resulted in an increase in the number of food service businesses worldwide in the last century [20]. The market size of the full-service restaurant industry worldwide is forecasted to reach $1.7 trillion USD by 2027, indicating a rapid growth [21]. Despite some concerns following the COVID-19 pandemic, UK survey data have shown consumer willingness to dine out, and a substantial growth of food-service businesses such as pubs, bars, and fast food restaurants [22]. However, since the incidence of foodborne illness, particularly associated with food consumed out of home, remains a concern worldwide, the increased number of studies on the topic of food safety in food service establishments may also be reflecting this concern. In this review the majority of studies originated from the United States (29%), Brazil (17%) and the United Kingdom (7%) (Table 1). Most importantly, as shown in this review, the publication of food handler food safety studies from 33 international countries indicates an international interest in the topic of food safety in the food service.

### 3.2 Types of food service settings in reviewed studies

Reviewed studies were conducted in food service settings of different types (Table 1). Restaurant establishments were investigated in the majority of studies (70%), followed by institutional food service facilities, e.g., those located in schools and universities (31%) (Table 1). The review demonstrated that quick-serve settings, such as takeaway and sandwich shop settings were least explored in the collated literature (17%). Importantly, in a third (33%) of reviewed studies the data collection was undertaken in multiple types of food service settings.

Restaurants are associated with the majority of foodborne outbreaks in food service sector [23]. This may indicate why such food service establishments have been most frequently investigated in the reviewed research. Additionally, due to the rapid market growth restaurants are often more abundant than other food service settings, and hence provide more access for data collection.

### 3.3 Research methods and measures in the reviewed studies

A variety of data collection methods were utilised in the reviewed studies (Table 2) including questionnaires, observational studies, interviews, microbiological assessment, and focus groups. Moreover, food handler cognitive measures and behavioural measures were assessed in the reviewed literature. Methodological approaches were evaluated and critically analysed, as described in the following sections.

**Table 2:** Methods of data collection in the reviewed peer-reviewed research (*n* = 118).

Method	*n* of studies	%^a^
**Questionnaire**	**94**	**80**
Paper-based offered on site	62	66
Face-to-face structured interview	19	20
Email/online	11	12
Combined methods	9	10
Telephone	3	3
Paper-based posted	2	2
**Observation**	**34**	**29**
Overt observation	29	85
Observation using a video footage	3	9
Covert observation	1	3
Notational analysis	1	3
**Qualitative interview**	**13**	**11**
Face-to-face	10	77
Telephone	3	23
**Microbiological assessment**	**12**	**10**
**Focus Group**	**4**	**3**

a Percentage in bold calculated out of the overall *n* = 118. Other percentages calculated out of the number of studies indicated in bold for that category, respectively.

### 3.4 Use of questionnaires in reviewed studies

In the area of food safety, a questionnaire (also known as a survey) is commonly used to gain information from the selected population sample about participants' knowledge of food handling practices, self-reported food safety practices, and participants’ cognitive factors, such as attitudes, perceptions, intention, beliefs, and motivations. The data gained from structured questionnaires are usually quantitative and standardized, allowing researchers to perform a quantitative analysis [24]. In this review, questionnaires were utilised in 80% of studies and thus were identified as the most utilised method of data collection concerning food safety in food service establishments. Importantly, more than half of the reviewed studies (55%) relied on questionnaires as a sole method for data collection.

Different approaches to data collection using a structured questionnaire were employed in the studies, as shown in Table 2. Paper-based questionnaires offered to respondents at food service establishments were most often used in the reviewed studies (66%). This approach may be beneficial because of convenience, since the questionnaire can be filled in and collected from the participants without the need to utilise postal services which could be costly. Hence, paper-based questionnaire posted to participants, or requiring the use of postal service to return the response was the least utilised method. Moreover, the physical presence of the researcher distributing the paper-based questionnaire may provide the sense of legitimacy of the research and promote better rapport between the researcher and the participant, thus potentially increasing the response rate [25].

It is important to highlight that in recent years technological progression may have promoted the use of online methods, since the Internet is a part of daily life [26]. Electronic survey administration has some benefits, such as cost-effectiveness, faster response and higher response rate [24,27]. On the other hand, it may also have a number of disadvantages, such as the potential for biases and for fraudulent or bot-generated survey responses, which may significantly affect the quality of collected data [25,28,29]. Therefore, all electronic questionnaires should be designed with appropriate measures built in to minimise such limitations. In this review the electronic administration methods were used in 12% of questionnaire-based studies, as seen in Table 2. Notably, the administration and completion of the online questionnaires among the food handlers may be more challenging to manage than the paper-based mode of questionnaire administration.

Some reviewed studies (*n* = 4) specified that the reason for utilising the face-to-face structured questionnaire was the fact that respondents had low level of literacy. Such participants may have difficulty understanding the questionnaire provided in written form and, therefore, a structured questionnaire delivered in verbally via an interviewer may ensure better understanding of questions and yield more reliable results.

Whilst any type of a questionnaire described (Table 2) allows the researcher to collect quantitative data from a population sample, using a questionnaire for data collection has limitations. Participants' responses may be a subject to social desirability which is defined as the “*tendency of some respondents to report an answer in a way they deem to be more socially acceptable than would be their "true" answer, in order to project a favourable image of themselves and to avoid receiving negative evaluations*” [30]. In fact, an in-person questionnaire administration method has been shown to increase the social desirability bias, due to the social interaction with the researcher [25]. It is also fair to suggest that a structured questionnaire delivered verbally may create an ‘interviewer bias’, where respondents may alter responses, depending on the perceptions about the interviewer's characteristics: tone of voice, neutrality towards specific responses, gender, and others [25,31].

### 3.5 Use of interviews and focus groups in reviewed studies

To obtain a qualitative in-depth understanding of respondents' feelings, opinions and attitudes towards a given complex topic researchers usually employ interview or focus group approach. Both interviews and focus groups can involve a series of open-ended questions which prompt participants to talk about the topic [32]. Interviews and focus groups rely on participants' willingness to share. Qualitative interviews can be conducted one-to-one, allowing participants to express their opinion to the interviewer. Focus groups involve a small homogenous group of participants to discuss a topic among themselves, whilst moderated and directed by the interviewer. The aim of a focus group is to create a comfortable and non-judgemental environment to encourage participants to share thoughts, feelings and opinions easily [33]. In the area of food safety these approaches have been often used to gain an in-depth information about participants' cognition, such as perception of risk of foodborne illness; participants' opinions about complex topics such as food safety culture; and about participants’ attitudes, such as: the attitude to food safety. When seeking answers about what participants think and why certain food safety practices are followed – interview and focus groups methods are particularly useful, providing an in-depth qualitative data. In this review 11% of studies utilised a qualitative interview approach for data collection, and only 3% of studies utilised focus groups (Table 2).

Importantly, these methods require specific skills, time management and resources, as opposed to faster and less costly methods, such as online questionnaire. Whilst providing an in-depth exploratory insight, these approaches have limitations, which need to be taken in consideration. For example, the validity of the obtained data may be undermined by the fact that self-reported accounts of behaviour rely on personal interpretation of actions. The accuracy of these accounts may depend on participants’ willingness to disclose information about the practices they perform, which may be substituted by an account of practices that are perceived to be more appropriate and are judged more favourably by the researcher. As previously mentioned, such tendency to describe what should be done instead of what was actually done, is referred to as social desirability bias [30]. Moreover, interviewee statements may be altered or misinterpreted due to interviewer bias [31,34]. The responses of focus group participants may be influenced by a particularly dominant individual in the group [35]. In such situations participants may knowingly provide false information according to how they want to be seen, but such data may lead to erroneous findings.

### 3.6 Use of observation in reviewed studies

Observation is a direct measure of behaviour enabling access to behaviour within context and over time [36]. Observational methods provide data that do not rely on participants' self-reports, and therefore can be more reliable. Observational methods can be: overt observation – where the researcher is present and the participants are aware that they are being observed; or covert observation – where the researcher is disguised and the participants are not aware that they are being observed, which can be undertaken using closed-circuit television (CCTV) cameras to obtain information about the actual behaviour of food handlers [37,38]. Moreover, methodological variations are utilised in observational studies, where structured and non-structured approach may be used. Structured observational studies involve a systematic record of participants’ actions, which is determined by a standardized and validated observational checklist. Another similar approach involves the use of notational analysis, where a predetermined coding schedule is utilised for recording observed sequence of actions. Such observational methods produce accurate quantifiable data which are used to determine specific behavioural aspects [36,39].

Whilst providing superior results, observational methods are not immune from potential bias; for example, the presence of a researcher in overt observation can cause reactivity bias known as the Hawthorne Effect [40,41], whereby the subject's behaviour may be altered as a result of being aware that they are being observed [42]. Thus, when utilising observational methods, reliability and validity of observational methods must be ensured. It is suggested that covert observation, using video cameras may reduce subject reactivity [43].

In this review only 29% of studies employed observational methods (Table 2). Most of the observations were direct (performed by a non-disguised observer) (85%), usually utilising a structured checklist or a form for the record of observed behaviour or environment (Table 2). Video observation was included in just 9% of observational studies. Covert observation (disguised from the participants) was used in one (*n* = 1) observational study assessing handwashing behaviour of food handling personnel and customers [44]. Notational analysis was utilised in one study (*n* = 1) for observation of cross-contamination practices of food handling personnel, proving to be an effective method of data collection [45].

### 3.7 Inclusion of microbiological assessment in reviewed studies

In food safety research the microbiological assessment of the food preparation environment, hands, food contact surfaces and end-product, enables a direct representation of the effectiveness of practices such as cleaning, sanitizing, and handwashing. Such approach provides data which do not depend on capturing participant cognition or on observation, and hence bypasses the possibility of social desirability bias, reactivity bias, or observer bias. When utilised in combination with other methods of data collection it has been reported to be very effective in determining the quality of food handler food safety practices which help avoid cross-contamination [44,46].

Microbiological assessment was implemented in 10% of studies in this review, two of which used it as a sole method of data collection [47,48]. Depending on the nature of the study, various microbiological tests for foodborne pathogens of interest were performed, as shown in Table 3. In the reviewed studies the microbiological contamination of hands was most frequently assessed (33%), followed by the microbiological assessment of food preparation environment (17%), and by the approach which combines the assessment of environment, hands, and food (17%).

**Table 3:** Types of microbiological assessment utilised in the reviewed studies that included microbiological assessment (*n* = 12).

% of studies	Environment	Hands	Food
33		✓	
17	✓		
17	✓	✓	✓
17	✓	✓	
8			✓
8	✓		✓

### 3.8 Use of mixed method approaches in reviewed studies

It is acknowledged that combining quantitative and qualitative methods in multidisciplinary research enables better understanding of a problem or a phenomenon through looking at it from different perspectives [49]. It must be highlighted that the selection of particular methods has to be based on the extent to which the combination of these methods can provide control over the assessment of measures and lead to authentic and valid results [50].

Just over a quarter (28%) of all studies in this review utilised mixed methods of data collection (Table 1). In food safety research, when utilising methods involving self-reported practices, such as questionnaires, interviews, and focus groups, it is important to obtain data which could help identify the possible discrepancies between the self-reported and the actual behaviour of food handlers and take in consideration the possibility of social desirability bias. Therefore, choosing suitable qualitative and quantitative methods can be challenging [51]. But provided that the chosen methodology is appropriate, the application of mixed method approach is central to triangulating the output, ensuring the validity of the findings and minimising the possibility of bias [52,53]. Given the merits, future food service sector food safety studies should triangulate research methods to obtain an in-depth understanding of food handler food safety.

3.9 Presentation of cognitive data in reviewed studies.

A considerable proportion of reviewed studies were focused on investigating cognitive factors relating to the food safety behaviour of food handlers in food service environments, such as food safety knowledge, attitudes, and perceptions of risk and responsibility when handling food. It is widely recognised that cognitive factors may be associated with food safety performance of food handlers [54].

Food safety knowledge refers to the awareness or information that food handlers gain through experience or training. More than a half of reviewed studies (58%) investigated food handlers’ knowledge. Without appropriate food safety knowledge, food handlers may not recognise the associated microbiological risks, why they exist, and how to mitigate them. In the study by De Boeck et al. (2017) knowledge was shown to be partially mediating food safety climate, compliance, participation and behaviour as a whole.

This review determined that ‘cross-contamination’ (45%) and ‘sources of foodborne illness’ (37%) were the most investigated aspects of food safety knowledge in the collated studies (Table 4). On the other hand, the knowledge of Hazard Analysis and Critical Control Point (HACCP) principles, which food handlers should be familiar with to work in food service establishment, was explored in only 10% of studies. Importantly, the knowledge of allergens and allergen control was investigated in only 3% of collated studies, highlighting the need for more research on this topic. Food handler knowledge of specific food safety practices was assessed in the reviewed research (Table 4). The knowledge of practices related to temperature control, such as cooking (36%) and refrigeration (34%) was most frequently assessed. However, the assessment of knowledge of food safety practices related to cross-contamination, such as handling raw meat (23%) and separation (18%) was lacking, indicating the need for inclusion of these aspects of food safety into the assessment of knowledge in the future.

**Table 4:** Food safety knowledge determined in reviewed food service food safety studies (*n* = 118).

	No of studies	% of studies
**Knowledge of specific attributes of food safety**		
Cross-contamination	53	45
Source of foodborne illness	44	37
Foodborne pathogens	39	33
Food poisoning	33	28
HACCP principles	12	10
Allergens	3	3
**Knowledge of appropriate food safety practices**		
Cooking	43	36
Refrigeration	40	34
Chilling	33	28
Cleaning	32	27
Freezing/Defrosting	32	27
Storage and date labelling	29	25
Handling meat and meat products (e.g., washing poultry)	27	23
Separation (cutting boards/zones in the kitchen)	21	18

Knowledge alone is not sufficient for improvement of food handlers' food safety practices [[55], [56], [57], [58], [59], [60]]. This is because other cognitive factors may influence food safety behaviour and are essential for transformation of knowledge into practice. For example, factors such as experiences, attitudes, emotions, and values have influence on individuals' risk perception and hence determine individuals’ risk mitigation behaviour [61].

Attitude is defined as a “*feeling or opinion about something or someone*” [62]. Food safety attitude can be described as food handlers' positive or negative evaluation of performing specific food safety practice. In this review 40% of studies reported assessment of food handler attitudes towards food safety. The majority of studies that explored food handler attitudes focused on the attitude towards ‘performing food safety practices’ (34%) and attitude towards ‘food safety in general’ (30%) (Table 5). A positive attitude was found to be important to process food safety knowledge transfer into practice [63,64]. Therefore, knowledgeable food handlers who recognise the importance of food safety implementation are suggested to be more likely to follow recommended food safety practices. However, whilst the transfer of adequate knowledge into practice may be aided by positive attitudes, it is not guaranteed [46,65]. Other cognitive factors may influence food handler compliance, and hence must also be considered to improve food safety.

**Table 5:** Food handler attitudes investigated in reviewed food service food safety studies (*n* = 118).

Attitude to	Count	% of studies
Performing food safety practices	40	34
Food safety in general	35	30
Company food safety policies	9	8
Management and supervisory staff	7	6
Hours and intensity of work	4	3
Not Specified	1	1

It is also important to use appropriate tools to assess knowledge and attitudes, ensuring that the data collected is specific and reliable. Although food handler knowledge is usually assessed via questionnaires, the content of the questions greatly varies in the reviewed research. It is notable that in several reviewed studies, the questions assessing food handler attitudes were quite generic and referred more to food handler knowledge of food safety. Oppenheim (1992) stressed that questions measuring attitudes have to be designed appropriately forming a scale and addressing not more than one issue, to allow the researcher to gain insight into participants’ tendency to respond to given issue in a certain way. Thus, there is a need for a standardized set of questions addressing food handler food safety knowledge and attitudes to food safety for a more robust assessment of cognition in future studies.

### 3.9 Presentation of behavioural data in reviewed studies

**Fig. 2:**
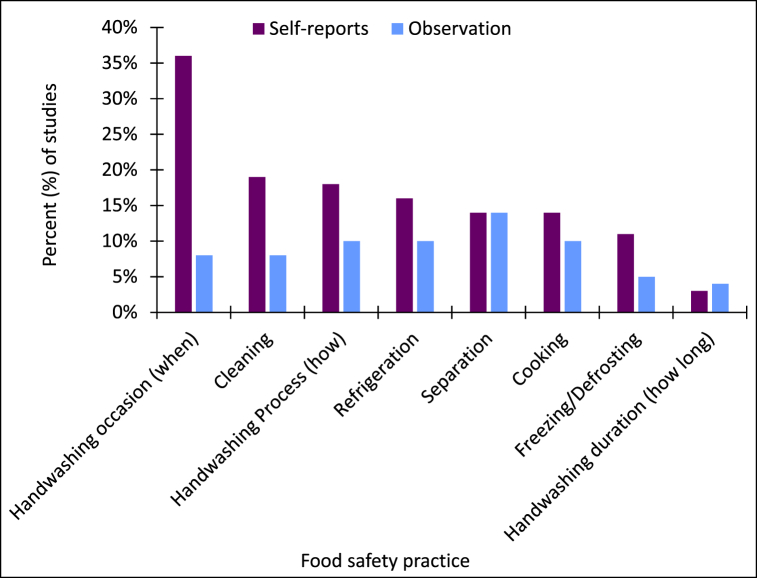
Self-reported food safety practices and observed food safety practices in the reviewed studies (*n* = 118).

Behavioural measures were also assessed in the reviewed literature, such as observed behaviour (24%) and self-reported food safety practices (41%) (Table 1). More than a third of food service food safety studies (41%) investigated food handler safe handling practices through assessment of self-reports. Self-reported implementation of hand hygiene at appropriate occasions (36%) was most frequently investigated, followed by self-reported cleaning practices (19%) (Fig. 2). Importantly, none of the studies addressed self-reported management of allergens when handling food or self-reported washing of raw meat or poultry in the food service environment.

Only 24% of studies reported obtaining observational data about food handler behaviour. In the reviewed studies, behaviours observed most frequently included separation of products, utensils and equipment to prevent cross-contamination (14%), handwashing process (10%), and practices related to safe cooking (10%) and refrigeration (10%). Cross-contamination, temperature control, handwashing and cleaning are identified as the areas of greatest concern in food service environments [3,66,67] and failure to implement these food safety practices appropriately may lead to increased risk of foodborne illness incident. Hence, it is of concern that there is a lack of behavioural data about food handlers’ implementation of such food safety practices, as shown in Fig. 2.

### 3.10 Use of mixed measure approaches in reviewed studies

**Fig. 3:**
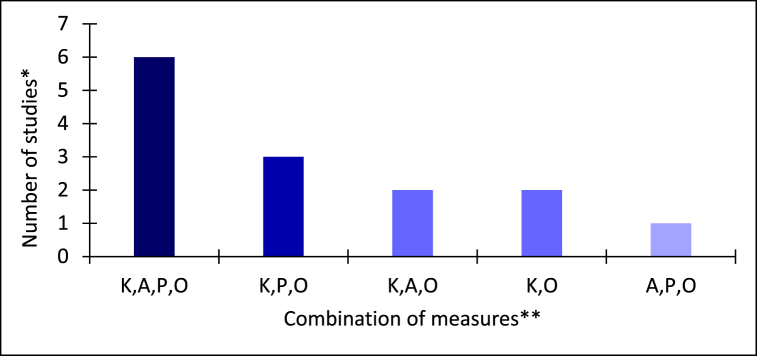
Reviewed studies investigating cognitive and behavioural measures in combination (*n* = 14).

To gain an accurate in-depth understanding of food handler food safety compliance it is important to consider both cognitive and behavioural findings that would reflect the complexity of food safety performance. It is suggested that mixed measure approach allows to view a specific phenomenon from different perspectives, which helps to represent a real-life scenario more accurately [68]. Only 14 studies in this review utilised measure combination approach (Fig. 3). A comprehensive assessment of cognitive measures (knowledge and attitudes) in combination with behavioural measures (self-reported practices and observed practices) was reported in only six reviewed research studies. It is suggested that the absence of mixed measure assessment may lead to erroneous or biased findings when food safety performance is considered. There is a need for a combined assessment of both food handler cognition and behaviour in the future food service food safety studies.

### 3.11 Study limitations and opportunities for future research

The research team would like to acknowledge several limitations of this review. Importantly, the data screening, extraction and analysis were performed by one reviewer. To ensure more rigorous results, it is suggested that multiple researchers are involved in the process of the future reviews.

It must be also acknowledged that the search criteria set for this review may have limited the number of collated studies, and that other peer-reviewed studies published in the specified period may be available to the readers. The limitation concerning the language is also of importance, as only the studies published in English were reviewed. Moreover, the research team note that the more recent studies, published after the 2021, are not included in this review. Thus, it is suggested that a future review including the primary studies after the year 2021 would be of value. This could be of particular importance, considering the possible impact of the COVID-19 pandemic on the food safety behaviour in food service and on the research methods, utilised for investigations.

Furthermore, the lack of critical appraisal of each individual study, included in this review, must be acknowledged as a limitation. However, it is important to bear in mind that the purpose of this review was to explore and synthesise the knowledge from all found peer-reviewed primary studies, selected using the chosen criteria, as described in Material and Methods.

Finally, although the lack of mixed method approach was identified in the reviewed studies, it must be considered that the researchers may be splitting bigger datasets of larger studies for the purpose of publication. In this case the published studies may contain the findings obtained only via one method or may be focused only on one specific measure of interest. Thus, it is suggested that it would be of benefit for larger projects to provide a combined account with cumulative findings from various methods, demonstrating the comparison or relationship between the behavioural and cognitive data.

## 4 Conclusion

Due to a rapid growth of food service industry internationally, prompted by consumers’ choice to eat out, much attention has been directed towards obtaining an in-depth understanding of food handler food safety behaviour in food service establishments in the last 20 years. The key findings from this review are as follows.

1. The majority of reviewed studies relied on a single method of data collection. Thus, there is a need for a reliable mixed method approach, involving observation, to confirm food safety compliance in food service establishments.
2. The reviewed studies separately investigated cognitive measures (knowledge and attitudes) and behavioural measures (observed behaviour and self-reports). However, studies assessing a combination of cognitive and behavioural measures in the food service are lacking.
3. There is an overall lack of data related to specific food handler practices, such as practices used when managing allergens and when handling raw meat products. Moreover, there is a need for observational studies assessing specific food safety practices such as handwashing, temperature control and cleaning and sanitizing in food service establishments.

The key findings from this review suggest the need to address the gaps in the future. It is recommended that the data about food handler food safety practices are best obtained through a combination of methods, assessing both cognitive and behavioural measures, and through the triangulation of the findings to gain a more accurate understanding of food handler food safety compliance in food service sector. Importantly, it is suggested that there is a need to standardize the questions used for the assessment of food handler food safety cognition to aid the knowledge synthesis and triangulation in future studies. Moreover, in the absence of many mixed method studies there is a need for researchers to explore the methods of triangulating findings obtained via single method from already existing peer-reviewed studies.

## Ethics statement

This study was reviewed and approved by Cardiff School of Sport and Health Sciences Ethics Committee at Cardiff Metropolitan University with the project reference: PGR-4434.

## Data availability statement

The review protocol, screening form, and data extraction form are available upon reasonable request to the corresponding author.

A reference list of all the reviewed studies is available as a Supplementary Material file.

## CRediT authorship contribution statement

**Veronika Bulochova:** Writing – review & editing, Writing – original draft, Project administration, Methodology, Investigation, Formal analysis, Conceptualization. **Ellen W. Evans:** Writing – review & editing, Supervision, Project administration, Funding acquisition, Conceptualization. **Claire Haven-Tang:** Writing – review & editing, Supervision, Conceptualization. **Elizabeth C. Redmond:** Writing – review & editing, Supervision, Project administration, Funding acquisition, Conceptualization.

## Declaration of competing interest

The authors declare that they have no known competing financial interests or personal relationships that could have appeared to influence the work reported in this paper.
