# Supplementary material for: Methods and measures in food service food safety research: A review of the published literature
Source: Heliyon. 2024 Feb 10;10(4):e25798. doi: 10.1016/j.heliyon.2024.e25798 (PMC10877249; doi:10.1016/j.heliyon.2024.e25798)
Supplement: Multimedia component 1 [file mmc1.pdf]

Methods and measures in food service food safety research: A review of the published literature.

Authors: Veronika Bulochova, Ellen W. Evans, Claire Haven-Tang, Elizabeth C. Redmond
January 2024.

Below are the references of collated studies (*n*=118) in alphabetical order.

Literature References

Ababio, P. F., Taylor, K. D. A., Daramola, B. A., & Swainson, M. (2016). Food law compliance in developed and developing countries: Comparing school kitchens in Lincolnshire-UK and Ashanti Region of Ghana. Food Control, 68, 167–173. https://doi.org/10.1016/j.foodcont.2016.03.023

Abdi, A. M., Amano, A., Abrahim, A., Getahun, M., Ababor, S., & Kumie, A. (2020). Food hygiene practices and associated factors among food handlers working in food establishments in the bole sub city, Addis Ababa, Ethiopia. Risk Management and Healthcare Policy, 13, 1861–1868. https://doi.org/10.2147/RMHP.S266342

Abdullah Sani, N., & Siow, O. N. (2014). Knowledge, attitudes and practices of food handlers on food safety in food service operations at the Universiti Kebangsaan Malaysia. Food Control, 37(1), 210–217. https://doi.org/10.1016/j.foodcont.2013.09.036

Abidin, U. F. U. Z., Arendt, S. W., & Strohbehn, C. H. (2013). Exploring the Culture of Food Safety: The Role of Organizational Influencers in Motivating Employees' Safe Food-Handling Practices. Journal of Quality Assurance in Hospitality and Tourism, 14(4), 321–343. https://doi.org/10.1080/1528008X.2013.802587

Adesokan, H. K., Akinseye, V. O., & Adesokan, G. A. (2015). Food safety training is associated with improved knowledge and behaviours among foodservice establishments workers. International Journal of Food Science, 2015. https://doi.org/10.1155/2015/328761

Akabanda, F., Hlortsi, E. H., & Owusu-Kwarteng, J. (2017). Food safety knowledge, attitudes and practices of institutional food-handlers in Ghana. BMC Public Health, 17(1), 1–9. https://doi.org/10.1186/s12889-016-3986-9

Al-Ghazali, M., Al-Bulushi, I., Al-Subhi, L., Rahman, M. S., & Al-Rawahi, A. (2020). Food Safety Knowledge and Hygienic Practices among Different Groups of Restaurants in Muscat, Oman. International Journal of Food Science, 2020. https://doi.org/10.1155/2020/8872981

Al-Kandari, D., Al-abdeen, J., & Sidhu, J. (2019). Food safety knowledge, attitudes and practices of food handlers in restaurants in Kuwait. Food Control, 103(April), 103–110. https://doi.org/10.1016/j.foodcont.2019.03.040

Al-Shabib, N. A., Mosilhey, S. H., & Husain, F. M. (2016). Cross-sectional study on food safety knowledge, attitude and practices of male food handlers employed in restaurants of King Saud University, Saudi Arabia. Food Control, 59, 212–217. https://doi.org/10.1016/j.foodcont.2015.05.002

Alemayehu, T., Aderaw, Z., Giza, M., & Diress, G. (2021). Food safety knowledge, handling practices and associated factors among food handlers working in food establishments in Debre Markos Town, Northwest Ethiopia, 2020: Institution-based cross-sectional study. Risk Management and Healthcare Policy, 14, 1155–1163. https://doi.org/10.2147/RMHP.S295974

Arendt, S. W., Paez, P., & Strohbehn, C. (2013). Food safety practices and managers' perceptions: A qualitative study in hospitality. International Journal of Contemporary Hospitality Management, 25(1), 124–139. https://doi.org/10.1108/09596111311290255

Ashraf, H. R. L., Atwood, S., Bloom, J., Blaise, D., & Salazar, J. (2008). Efficacy of HACCP-based food handling training program for front-line foodservice workers: A report on a collaborative work between regulatory agencies and academia. Journal of Culinary Science and Technology, 6(1), 63–76. https://doi.org/10.1080/15428050701884204

Azanaw, J., Gebrehiwot, M., & Dagne, H. (2019). Factors associated with food safety practices among food handlers: Facility-based cross-sectional study. BMC Research Notes, 12(1), 10–15. https://doi.org/10.1186/s13104-019-4702-5

Bailey, S., Albardiaz, R., Frew, A. J., & Smith, H. (2011). Restaurant staff's knowledge of anaphylaxis and dietary care of people with allergies. Clinical and Experimental Allergy, 41(5), 713–717. https://doi.org/10.1111/j.1365-2222.2011.03748.x

Barbosa, F. M., de Souza, C. V. S., Ribeiro, E. S., de Azevedo, P. R. M., da Silva Chaves Damasceno, K. S. F., & Mont'Alverne Jucá Seabra, L. (2021). Do as I say or as I do? Food handler's knowledge on good handling practices and evaluation of hygienic–sanitary conditions in hospital foodservices. Journal of Food Safety, 41(1). https://doi.org/10.1111/jfs.12869

Betts, K. R., & Hinsz, V. B. (2015). Mindful Attention and Awareness Predict Self-Reported Food Safety Practices in the Food Service Industry. Current Psychology, 34(2), 191–206. https://doi.org/10.1007/s12144-014-9251-4

Bolton, D. J., Meally, A., Blair, I. S., McDowell, D. A., & Cowan, C. (2008). Food safety knowledge of head chefs and catering managers in Ireland. Food Control, 19(3), 291–300. https://doi.org/10.1016/j.foodcont.2007.04.006

Bou-Mitri, C., Mahmoud, D., El Gerges, N., & Jaoude, M. A. (2018). Food safety knowledge, attitudes and practices of food handlers in Lebanese hospitals: A cross-sectional study. Food Control, 94(April), 78–84. https://doi.org/10.1016/j.foodcont.2018.06.032

Brannon, L. A., York, V. K., Roberts, K. R., Shanklin, C. W., & Howells, A. D. (2009). Appreciation of food safety practices based on level of experience. Journal of Foodservice Business Research, 12(2), 134–154. https://doi.org/10.1080/15378020902910462

Casolani, N., & Del Signore, A. (2016). Managers' opinions of factors in Italian hotel/restaurant/café (HoReCa) sector. British Food Journal, 118(5), 1195–1207.

Chapman, B., Eversley, T., Fillion, K., MacLaurin, T., & Powell, D. (2010). Assessment of food safety practices of food service food handlers (risk assessment data): Testing a communication intervention (evaluation of tools). Journal of Food Protection, 73(6), 1101–1107. https://doi.org/10.4315/0362-028X-73.6.1101

Cho, S., Hertzman, J., Erdem, M., & Garriott, P. O. (2013). A Food Safety Belief Model for Latino(A) Employees in Foodservice. Journal of Hospitality and Tourism Research, 37(3), 330–348. https://doi.org/10.1177/1096348012436378

Choi, J., Norwood, H., Seo, S., Sirsat, S. A., & Neal, J. (2016). Evaluation of food safety related behaviors of retail and food service employees while handling fresh and fresh-cut leafy greens. Food Control, 67, 199–208. https://doi.org/10.1016/j.foodcont.2016.02.044

Clark, J., Crandall, P., & Reynolds, J. (2019). Exploring the influence of food safety climate indicators on handwashing practices of restaurant food handlers. International Journal of Hospitality Management, 77(January 2018), 187–194. https://doi.org/10.1016/j.ijhm.2018.06.029

Clayton, D. A., & Griffith, C. J. (2004). Observation of food safety practices in catering using notational analysis. British Food Journal, 106(3), 211–227. https://doi.org/10.1108/00070700410528790

Clayton, M. L., Smith, K. C., Neff, R. A., Pollack, K. M., & Ensminger, M. (2015). Listening to food workers: Factors that impact proper health and hygiene practice in food service. International Journal of Occupational and Environmental Health, 21(4), 314–327. https://doi.org/10.1179/2049396715Y.0000000011

Cui, B., Li, S. Y., Wang, L. D. L., Chen, X., Ke, J., & Tian, Y. (2021). Hand hygiene knowledge and self-reported hand washing behaviors among restaurant kitchen chefs in Jiangsu Province, China. International Journal of Environmental Research and Public Health, 18(4), 1–14. https://doi.org/10.3390/ijerph18042149

da Cunha, D. T., Braga, A. R. C., Passos, E. de C., Stedefeldt, E., & de Rosso, V. V. (2015). The existence of optimistic bias about foodborne disease by food handlers and its association with training participation and food safety performance. Food Research International, 75, 27–33. https://doi.org/10.1016/j.foodres.2015.05.035

da Cunha, D. T., de Rosso, V. V., Pereira, M. B., & Stedefeldt, E. (2019). The differences between observed and self-reported food safety practices: A study with food handlers using structural equation modeling. Food Research International, 125(May). https://doi.org/10.1016/j.foodres.2019.108637

da Cunha, D. T., Stedefeldt, E., & de Rosso, V. V. (2012). Perceived risk of foodborne disease by school food handlers and principals: The influence of frequent training. Journal of Food Safety, 32(2), 219–225. https://doi.org/10.1111/j.1745-4565.2012.00371.x

da Cunha, D. T., Stedefeldt, E., & de Rosso, V. V. (2014). The role of theoretical food safety training on Brazilian food handlers' knowledge, attitude and practice. Food Control, 43, 167–174. https://doi.org/10.1016/j.foodcont.2014.03.012

da Vitória, A. G., de Souza Couto Oliveira, J., de Almeida Pereira, L. C., de Faria, C. P., & de São José, J. F. B. (2021). Food safety knowledge, attitudes and practices of food handlers: A cross-sectional study in school kitchens in Espírito Santo, Brazil. BMC Public Health, 21(1), 1–10. https://doi.org/10.1186/s12889-021-10282-1

de Andrade, M. L., Rodrigues, R. R., Antongiovanni, N., & da Cunha, D. T. (2019). Knowledge and risk perceptions of foodborne disease by consumers and food handlers at restaurants with different food safety profiles. Food Research International, 121(January), 845–853. https://doi.org/10.1016/j.foodres.2019.01.006

de Andrade, M. L., Stedefeldt, E., Zanin, L. M., & da Cunha, D. T. (2020). Food safety culture in food services with different degrees of risk for foodborne diseases in Brazil. Food Control, 112(February), 107152. https://doi.org/10.1016/j.foodcont.2020.107152

de Andrade, M. L., Stedefeldt, E., Zanin, L. M., Zanetta, L. D., & da Cunha, D. T. (2021). Unveiling the food safety climate's paths to adequate food handling in the hospitality industry in Brazil. International Journal of Contemporary Hospitality Management, 33(3), 873–892. https://doi.org/10.1108/IJCHM-09-2020-1030

de Freitas, R. S. G., da Cunha, D. T., & Stedefeldt, E. (2019). Food safety knowledge as gateway to cognitive illusions of food handlers and the different degrees of risk perception. Food Research International, 116(July 2018), 126–134. https://doi.org/10.1016/j.foodres.2018.12.058

DiPietro, R. B., Harris, K., & Jin, D. (2020). Employed in the foodservice industry: likelihood of intervention with food safety threats. International Hospitality Review, 34(2), 243–262. https://doi.org/10.1108/ihr-07-2020-0021

Disanto, C., Celano, G., Dambrosio, A., Quaglia, N. C., Bozzo, G., Tritto, A., & Celano, G. V. (2020). Food safety in collective catering: Knowledge, attitudes and correct application of GHP/GMP knowledge among foodservice workers. Italian Journal of Food Safety, 9(4), 201–205. https://doi.org/10.4081/ijfs.2020.8453

do Prado, D. B., Bettoni, A. P., Correa, V. A., de Abreu Filho, B. A., Garcia, L. B., Tognim, M. C. B., & Cardoso, C. L. (2015). Practice of hand hygiene in a university dining facility. Food Control, 57, 35–40. https://doi.org/10.1016/j.foodcont.2015.03.024

Ellis, J. D., Arendt, S. W., Strohbehn, C. H., Meyer, J., & Paez, P. (2010). Varying influences of motivation factors on employees' likelihood to perform safe food handling practices because of demographic differences. Journal of Food Protection, 73(11), 2065–2071. https://doi.org/10.4315/0362-028X-73.11.2065

Elobeid, T., Savvaidis, I., & Ganji, V. (2019). Impact of food safety training on the knowledge, practice, and attitudes of food handlers working in fast-food restaurants. British Food Journal, 121(4), 937–949. https://doi.org/10.1108/BFJ-01-2019-0066

Faour-Klingbeil, D., Kuri, V., & Todd, E. (2015). Investigating a link of two different types of food business management to the food safety knowledge, attitudes and practices of food handlers in Beirut, Lebanon. Food Control, 55, 166–175. https://doi.org/10.1016/j.foodcont.2015.02.045

Frash, R. E., & Maclaurin, T. (2010). Restaurant food safety: The influence of employee outlooks on transfer of training. International Journal of Hospitality and Tourism Administration, 11(4), 328–346. https://doi.org/10.1080/15256480.2010.518523

Garayoa, R., Abundancia, C., Díez-Leturia, M., & Vitas, A. I. (2017). Essential tools for food safety surveillance in catering services: On-site inspections and control of high risk cross-contamination surfaces. Food Control, 75, 48–54. https://doi.org/10.1016/j.foodcont.2016.12.032

Garayoa, R., Vitas, A. I., Díez-Leturia, M., & García-Jalón, I. (2011). Food safety and the contract catering companies: Food handlers, facilities and HACCP evaluation. Food Control, 22(12), 2006–2012. https://doi.org/10.1016/j.foodcont.2011.05.021

Garayoa, R., Yánez, N., Díez-Leturia, M., Bes-Rastrollo, M., & Vitas, A. I. (2016). Evaluation of Prerequisite Programs Implementation and Hygiene Practices at Social Food Services through Audits

and Microbiological Surveillance. Journal of Food Science, 81(4), M921–M927. https://doi.org/10.1111/1750-3841.13258

Ghezzi, S., & Ayoun, B. (2013). Food safety in the US catering industry: Empirical findings. International Journal of Contemporary Hospitality Management, 25(3), 365–382. https://doi.org/10.1108/09596111311311026

Gomes, C. C. B., Lemos, G. F. C., Silva, M. C., Hora, I. M. C., & Cruz, A. G. (2014). Training of Food Handlers in a Hotel: Tool for Promotion of the Food Safety. Journal of Food Safety, 34(3), 218–223. https://doi.org/10.1111/jfs.12116

Gruenfeldova, J., Domijan, K., & Walsh, C. (2019). A study of food safety knowledge, practice and training among food handlers in Ireland. Food Control, 105(January), 131–140. https://doi.org/10.1016/j.foodcont.2019.05.023

Guchait, P., Neal, J. A., & Simons, T. (2016). Reducing food safety errors in the United States: Leader behavioral integrity for food safety, error reporting, and error management. International Journal of Hospitality Management, 59, 11–18. https://doi.org/10.1016/j.ijhm.2016.08.008

Hamed, A., & Mohammed, N. (2020). Food safety knowledge, attitudes and self-reported practices among food handlers in Sohag governorate, Egypt. Eastern Mediterranean Health Journal, 26(4), 374–381. https://doi.org/10.26719/emhj.19.047

Harris, K. J., DiPietro, R. B., Line, N. D., & Murphy, K. S. (2019). Restaurant employees and food safety compliance: motivation comes from within. Journal of Foodservice Business Research, 22(1), 98–115. https://doi.org/10.1080/15378020.2018.1547037

Harris, K. J., Murphy, K. S., DiPietro, R. B., & Line, N. D. (2017). The antecedents and outcomes of food safety motivators for restaurant workers: An expectancy framework. International Journal of Hospitality Management, 63, 53–62. https://doi.org/10.1016/j.ijhm.2017.02.004

Harris, K., Taylor, S., & DiPietro, R. B. (2021). Antecedents and outcomes of restaurant employees' food safety intervention behaviors. International Journal of Hospitality Management, 94(December 2020), 102858. https://doi.org/10.1016/j.ijhm.2021.102858

Hashanuzzaman, M., Bhowmik, S., Rahman, M. S., Zakaria, M. U. M. A., Voumik, L. C., & Mamun, A. Al. (2020). Assessment of food safety knowledge, attitudes and practices of fish farmers and restaurants food handlers in Bangladesh. Heliyon, 6(11), 0–7. https://doi.org/10.1016/j.heliyon.2020.e05485

Hedeen, N., Reimann, D., & Everstine, K. (2016). Microwave cooking practices in Minnesota food service establishments. Journal of Food Protection, 79(3), 507–511. https://doi.org/10.4315/0362-028X.JFP-15-333

Her, E., Behnke, C., & Almanza, B. (2019). Does a water flow timer improve food handler hand washing practices in food service establishments? The effects of passive and indirect interventions. Journal of Environmental Health, 81(8), 8–13.

Johnson, L., Shin, J. H., Feinstein, A. H., & Mayer, K. J. (2003). Validating a food safety instrument: Measuring food safety knowledge and attitudes of restaurant employees. Journal of Foodservice Business Research, 6(2), 49–65. https://doi.org/10.1300/J369v06n02_05

Kılıçhan, R., Çalhan, H., & Umur, M. (2020). Food safety attitudes and practices of chefs in Cappadocia region, Turkey. Journal of Foodservice Business Research, 23(3), 193–215. https://doi.org/10.1080/15378020.2020.1718401

Ko, W. H. (2013). The relationship among food safety knowledge, attitudes and self-reported HACCP practices in restaurant employees. Food Control, 29(1), 192–197. https://doi.org/10.1016/j.foodcont.2012.05.076

Ko, W. H., & Kang, H. yu. (2019). Effect of leadership style and organizational climate on employees' food safety and hygiene behaviors in the institutional food service of schools. Food Science and Nutrition, 7(6), 2131–2143. https://doi.org/10.1002/fsn3.1056

Kramer, J., & Scott, W. G. (2004). Food safety knowledge and practices in ready-to-eat food establishments. International Journal of Environmental Health Research, 14(5), 343–350. https://doi.org/10.1080/09603120400004022

Kwol, V. S., Avci, T., Eluwole, K. K., & Dalhatu, A. (2020). Food safety knowledge and hygienic-sanitary control: A needed company for public well-being. Journal of Public Affairs, 20(3). https://doi.org/10.1002/pa.2067

Lee, H. K., Abdul Halim, H., Thong, K. L., & Chai, L. C. (2017). Assessment of food safety knowledge, attitude, self-reported practices, and microbiological hand hygiene of food handlers. International Journal of Environmental Research and Public Health, 14(1). https://doi.org/10.3390/ijerph14010055

Lee, J. E., Almanza, B. A., Jang, S. C., Nelson, D. C., & Ghiselli, R. F. (2013). Does transformational leadership style influence employees' attitudes toward food safety practices? International Journal of Hospitality Management, 33(1), 282–293. https://doi.org/10.1016/j.ijhm.2012.09.004

Liggans, G. L., Boyer, M. S., Williams, L. B., Destromp, K. W., & Hoang, S. T. (2019). Food safety management systems, certified food protection managers, and compliance with food safety practices associated with the control of Listeria monocytogenes in foods at restaurants. Journal of Food Protection, 82(7), 1116–1123. https://doi.org/10.4315/0362-028X.JFP-18-532

Lin, N., & Paez, P. (2020). Leading by example: A three-wave sequential mixed method food safety study. International Journal of Hospitality Management, 87(July 2019), 102463. https://doi.org/10.1016/j.ijhm.2020.102463

liu, S., liu, Z., Zhang, H., Lu, L., Liang, J., & Huang, Q. (2015). Knowledge, attitude and practices of food safety amongst food handlers in the coastal resort of Guangdong, China. Food Control, 47, 457–461. https://doi.org/10.1016/j.foodcont.2014.07.048

Lynch, R. A., Elledge, B. L., Griffith, C. C., & Boatright, D. T. (2003). A Comparison of Food Safety Knowledge among Restaurant Managers, by Source of Training and Experience, in Oklahoma County, Oklahoma. Journal of Environmental Health, 66(2), 9–14.

Machado Dr., M. G., Monego, E. T., & Campos, M. R. H. (2014). Risk perception of food safety by school food-handlers. Journal of Health, Population and Nutrition, 32(1), 19–27. https://doi.org/10.3329/jhpn.v32i1.2464

Manes, M. R., Kuganantham, P., Jagadeesan, M., Laxmidevi, M., & Dworkin, M. S. (2016). A Step Towards Improving Food Safety in India: Determining Baseline Knowledge and Behaviors Among Restaurant Food Handlers in Chennai. Journal of Environmental Health, 78(6), 18–26.

Maughan, C., Chambers, E., & Godwin, S. (2017). Food safety behaviors observed in celebrity chefs across a variety of programs. Journal of Public Health (United Kingdom), 39(1), 105–112. https://doi.org/10.1093/pubmed/fdw026

McIntyre, L., Vallaster, L., Wilcott, L., Henderson, S. B., & Kosatsky, T. (2013). Evaluation of food safety knowledge, attitudes and self-reported hand washing practices in FOODSAFE trained and untrained food handlers in British Columbia, Canada. Food Control, 30(1), 150–156. https://doi.org/10.1016/j.foodcont.2012.06.034

Moghnia, O. H., Rotimi, V. O., & Al-Sweih, N. A. (2021). Evaluating food safety compliance and hygiene practices of food handlers working in community and healthcare settings in Kuwait. International Journal of Environmental Research and Public Health, 18(4), 1–12. https://doi.org/10.3390/ijerph18041586

Mukhtar, M. A., Tamby Chik, C., Ariffin, H. F., & Abdul Rahman, A. R. (2021). Job competency of foreign workers' in shah alam restaurants: how does knowledge, attitude, practices and training effectiveness influence it? Journal of Foodservice Business Research, 00(00), 1–21. https://doi.org/10.1080/15378020.2021.1934249

Mulugeta, K., & Bayeh, A. (2012). The sanitary conditions of food service establishments and food safety knowledge and practices of food handlers in Bahir Dar town. Ethiopian Journal of Health Sciences, 22(1), 27–35. https://doi.org/10.4314/EJHS.V22I1

Ncube, F., Kanda, A., Chijokwe, M., Mabaya, G., & Nyamugure, T. (2020). Food safety knowledge, attitudes and practices of restaurant food handlers in a lower-middle-income country. Food Science and Nutrition, 8(3), 1677–1687. https://doi.org/10.1002/fsn3.1454

Nik Husain, N. R., Wan Muda, W. M., Noor Jamil, N. I., Nik Hanafi, N. N., & Abdul Rahman, R. (2016). Effect of food safety training on food handlers' knowledge and practices: A randomized controlled trial. British Food Journal, 118(4), 795–808. https://doi.org/10.1108/BFJ-08-2015-0294

Odonkor, S. T., & Odonkor, C. J. A. (2020). An Assessment of Food Safety Knowledge and Practices in the Ghanaian Hospitality Industry. Journal of Food Quality, 2020. https://doi.org/10.1155/2020/5618492

Osaili, T. M., Abu Jamous, D. O., Obeidat, B. A., Bawadi, H. A., Tayyem, R. F., & Subih, H. S. (2013). Food safety knowledge among food workers in restaurants in Jordan. Food Control, 31(1), 145–150. https://doi.org/10.1016/j.foodcont.2012.09.037

Osaili, T. M., Obeidat, B. A., Hajeer, W. A., & Al-Nabulsi, A. A. (2017). Food safety knowledge among food service staff in hospitals in Jordan. Food Control, 78, 279–285. https://doi.org/10.1016/j.foodcont.2017.02.057

Ozilgen, S. (2010). Application of failure mode and effect analysis model to foodservice systems operated by chefs in practice and by chefs from a culinary school in Turkey. Journal Fur Verbraucherschutz Und Lebensmittelsicherheit, 5(3), 333–343. https://doi.org/10.1007/s00003-010-0626-7

Park, S. H., Kwak, T. K., & Chang, H. J. (2010). Evaluation of the food safety training for food handlers in restaurant operations. Nutrition Research and Practice, 4(1), 58–68. https://doi.org/10.4162/nrp.2010.4.1.58

Parry-Hanson Kunadu, A., Ofosu, D. B., Aboagye, E., & Tano-Debrah, K. (2016). Food safety knowledge, attitudes and self-reported practices of food handlers in institutional foodservice in Accra, Ghana. Food Control, 69, 324–330. https://doi.org/10.1016/j.foodcont.2016.05.011

Pichler, J., Ziegler, J., Aldrian, U., & Allerberger, F. (2014). Evaluating levels of knowledge on food safety among food handlers from restaurants and various catering businesses in Vienna, Austria 2011/2012. Food Control, 35(1), 33–40. https://doi.org/10.1016/j.foodcont.2013.06.034

Pragle, A. S., Harding, A. K., & Mack, J. C. (2007). Food workers' perspectives on handwashing behaviors and barriers in the restaurant environment. Journal of Environmental Health, 70(2), 36.

Rebouças, L. T., Santiago, L. B., Martins, L. S., Rios Menezes, A. C., Araújo, M. da P. N., & Almeida, R. C. de C. (2017). Food safety knowledge and practices of food handlers, head chefs and managers in hotels' restaurants of Salvador, Brazil. Food Control, 73, 372–381. https://doi.org/10.1016/j.foodcont.2016.08.026

Roberts, K. R., & Barrett, B. B. (2011). Restaurant managers' beliefs about food safety training: An application of the theory of planned behavior. Journal of Foodservice Business Research, 14(3), 206–225. https://doi.org/10.1080/15378020.2011.594379

Rodrigues, K. L., Eves, A., das Neves, C. P., Souto, B. K., & dos Anjos, S. J. G. (2020). The role of Optimistic Bias in safe food handling behaviours in the food service sector. Food Research International, 130, 108732. https://doi.org/10.1016/j.foodres.2019.108732

Roseman, M. G., Mathe-Soulek, K., & Krawczyk, M. (2017). The effect of psychological empowerment climate on restaurant food safety, food quality, and financial performance. Journal of Human Resources in Hospitality and Tourism, 16(2), 137–152. https://doi.org/10.1080/15332845.2016.1202726

Rossi, M. de S. C., Stedefeldt, E., da Cunha, D. T., & de Rosso, V. V. (2017). Food safety knowledge, optimistic bias and risk perception among food handlers in institutional food services. Food Control, 73, 681–688. https://doi.org/10.1016/j.foodcont.2016.09.016

Salazar, J., Ashraf, H. R., Tcheng, M., & Antun, J. (2006). Food service employee satisfaction and motivation and the relationship with learning food safety. Journal of Culinary Science and Technology, 4(2–3), 93–108. https://doi.org/10.1300/J385v04n02_07

Sanlier, N., Cömert, M., & Durlu-Özkaya, F. (2010). Hygiene perception: Condition of hotel kitchen staffs in ankara, Turkey. Journal of Food Safety, 30(2), 415–431. https://doi.org/10.1111/j.1745-4565.2010.00216.x

Santos, M. J., Nogueira, J. R., Patarata, L., & Mayan, O. (2008). Knowledge levels of food handlers in Portuguese school canteens and their self-reported behaviour towards food safety. International Journal of Environmental Health Research, 18(6), 387–401. https://doi.org/10.1080/09603120802100212

Seaman, P., & Eves, A. (2008). Food hygiene training in small to medium-sized care settings. International Journal of Environmental Health Research, 18(5), 365–374. https://doi.org/10.1080/09603120802272193

Seaman, P., & Eves, A. (2010). Efficacy of the theory of planned behaviour model in predicting safe food handling practices. Food Control, 21(7), 983–987. https://doi.org/10.1016/j.foodcont.2009.12.012

Sibanyoni, J. J., Tshabalala, P. A., & Tabit, F. T. (2017). Food safety knowledge and awareness of food handlers in school feeding programmes in Mpumalanga, South Africa. Food Control, 73, 1397–1406. https://doi.org/10.1016/j.foodcont.2016.11.001

Silva, C. T., Hakim, M. P., Zanetta, L. D. A., Pinheiro, G. S. D. D., Gemma, S. F. B., & da Cunha, D. T. (2021). Burnout and food safety: Understanding the role of job satisfaction and menu complexity in foodservice. International Journal of Hospitality Management, 92(May 2020). https://doi.org/10.1016/j.ijhm.2020.102705

Sirichokchatchawan, W., Taneepanichskul, N., & Prapasarakul, N. (2021). Predictors of knowledge, attitudes, and practices towards food safety among food handlers in Bangkok, Thailand. Food Control, 126(February), 108020. https://doi.org/10.1016/j.foodcont.2021.108020

Smith, L., Sirsat, S. A., & Neal, J. A. (2014). Does food safety training for non-profit food service volunteers improve food safety knowledge and behavior? Food Protection Trends, 34(3), 156–165.

Soares, L. S., Almeida, R. C. C., Cerqueira, E. S., Carvalho, J. S., & Nunes, I. L. (2012). Knowledge, attitudes and practices in food safety and the presence of coagulase-positive staphylococci on hands of food handlers in the schools of Camaçari, Brazil. Food Control, 27(1), 206–213. https://doi.org/10.1016/j.foodcont.2012.03.016

Sobaih, A. E. E. (2011). Half job-half training? Management perceptions of part-time employee training in the hospitality industry. Journal of Human Resources in Hospitality and Tourism, 10(4), 400–420. https://doi.org/10.1080/15332845.2011.588563

Soon, J. M. (2019). Finger licking good? An observational study of hand hygiene practices of fast food restaurant employees and consumers. British Food Journal, 121(3), 697–710. https://doi.org/10.1108/BFJ-07-2018-0420

Souza, C. V. S. de, Azevedo, P. R. M. de, & Seabra, L. M. A. J. (2018). Food safety in Brazilian popular public restaurants: Food handlers' knowledge and practices. Journal of Food Safety, 38(5), 1–9. https://doi.org/10.1111/jfs.12512

Souza, P. A., & Santos, D. A. (2009). Microbiological risk factors associated with food handlers in elementary schools from Brazil. Journal of Food Safety, 29(3), 424–429. https://doi.org/10.1111/j.1745-4565.2009.00166.x

Taha, S., Osaili, T. M., Saddal, N. K., Al-Nabulsi, A. A., Ayyash, M. M., & Obaid, R. S. (2020). Food safety knowledge among food handlers in food service establishments in United Arab Emirates. Food Control, 110(July 2019), 106968. https://doi.org/10.1016/j.foodcont.2019.106968

Taha, S., Osaili, T. M., Vij, A., Albloush, A., & Nassoura, A. (2020). Structural modelling of relationships between food safety knowledge, attitude, commitment and behavior of food handlers in restaurants in Jebel Ali Free Zone, Dubai, UAE. Food Control, 118(February), 107431. https://doi.org/10.1016/j.foodcont.2020.107431

Tan, S. L., Bakar, F. A., Abdul Karim, M. S., Lee, H. Y., & Mahyudin, N. A. (2013). Hand hygiene knowledge, attitudes and practices among food handlers at primary schools in Hulu Langat district, Selangor (Malaysia). Food Control, 34(2), 428–435. https://doi.org/10.1016/j.foodcont.2013.04.045

Tang, C. H., & Fong, U. W. (2004). A survey of food hygiene knowledge and attitudes among Chinese food handlers in Fong Song Tong district. Asia-Pacific Journal of Public Health, 16(2), 121–125. https://doi.org/10.1177/101053950401600208

Taylor, E., Al Yousuf, M., Nassar, E. S., Saleh, M., & Philip, J. (2015). The small business dilemma: Understanding and reacting to the unique requirements of Abu Dhabi small businesses in achieving food safety standards. Worldwide Hospitality and Tourism Themes, 7(1), 50–62. https://doi.org/10.1108/WHATT-12-2014-0040

Taylor, J. Z. (2008). HACCP for the hospitality industry: A psychological model for success. International Journal of Contemporary Hospitality Management, 20(5), 508–523. https://doi.org/10.1108/09596110810881445

Teffo, L. A., & Tabit, F. T. (2020). An assessment of the food safety knowledge and attitudes of food handlers in hospitals. BMC Public Health, 20(1), 1–12. https://doi.org/10.1186/s12889-020-8430-5

Tran, B. X., Do, H. T., Nguyen, L. T., Boggiano, V., Le, H. T., Thi Le, X. T., Trinh, N. B., Nam Do, K., Nguyen, C. T., Nguyen, T. T., Dang, A. K., Mai, H. T., Nguyen, L. H., Than, S., & Latkin, C. A. (2018). Evaluating food safety knowledge and practices of food processors and sellers working in food facilities in Hanoi, Vietnam. Journal of Food Protection, 81(4), 646–652. https://doi.org/10.4315/0362-028X.JFP-17-161

Wham, C. A., & Sharma, K. M. (2014). Knowledge of café and restaurant managers to provide a safe meal to food allergic consumers. Nutrition and Dietetics, 71(4), 265–269. https://doi.org/10.1111/1747-0080.12104

Woh, P. Y., Thong, K. L., Behnke, J. M., Lewis, J. W., & Mohd Zain, S. N. (2016). Evaluation of basic knowledge on food safety and food handling practices amongst migrant food handlers in Peninsular Malaysia. Food Control, 70, 64–73. https://doi.org/10.1016/j.foodcont.2016.05.033

York, V. K., Brannon, L. A., Shanklin, C. W., Roberts, K. R., Barrett, B. B., & Howells, A. D. (2009). Intervention improves restaurant employees' food safety compliance rates. International Journal of Contemporary Hospitality Management, 21(4), 459–478. https://doi.org/10.1108/09596110910955703

Yu, H., Ma, J., Liu, P., & Sirsat, S. A. (2020). Investigating the effects of explanatory-based food safety training: A model of domain knowledge theory perspective. International Journal of Hospitality Management, 91(August), 102688. https://doi.org/10.1016/j.ijhm.2020.102688

Yu, H., Neal, J., Dawson, M., & Madera, J. M. (2018). Implementation of Behavior-Based Training Can Improve Food Service Employees' Handwashing Frequencies, Duration, and Effectiveness. Cornell Hospitality Quarterly, 59(1), 70–77. https://doi.org/10.1177/1938965517704370
